# The Use of Smartphone-Based Triage to Reduce the Rate of Outpatient Error Registration: Cross-Sectional Study

**DOI:** 10.2196/15313

**Published:** 2019-11-11

**Authors:** Wanhua Xie, Xiaojun Cao, Hongwei Dong, Yu Liu

**Affiliations:** 1 Outpatient Department Guangzhou Women and Children's Medical Center Guangzhou Medical University Guangzhou China; 2 Department of Science Education and Data Management Guangzhou Women and Children's Medical Center, Guangzhou Medical University Guangzhou China; 3 Hospital Office Guangzhou Women and Children's Medical Center Guangzhou Medical University Guangzhou China

**Keywords:** smartphone, triage, outpatients, personal satisfaction

## Abstract

**Background:**

In many clinics, patients now have the option to make Web-based appointments but doing so according to their own judgment may lead to wrong registration and delayed medical services. We hypothesized that smartphone-based triage in outpatient services is superior to Web-based self-appointment registration guided by the medical staff.

**Objective:**

This study aimed to investigate smartphone-based triage in outpatient services compared with Web-based self-appointment registration and to provide a reference for improving outpatient care under appointment registration.

**Methods:**

The following parameters in Guangzhou Women and Children’s Medical Center were analyzed: wrong registration rate, the degree of patient satisfaction, outpatient visits 6 months before and after smartphone-based triage, queries after smartphone-based triage, number of successful registrations, inquiry content, and top 10 recommended diseases and top 10 recommended departments after queries.

**Results:**

Smartphone-based triage showed significant effects on average daily queries, which accounted for 16.15% (1956/12,112) to 29.46% (3643/12,366) of daily outpatient visits. The average daily successful registration after queries accounted for 56.14% (1101/1961) to 60.92% (1437/2359) of daily queries and 9.33% (1130/12,112) to 16.83% (2081/12,366) of daily outpatient visits. The wrong registration rate after smartphone-based triage was reduced from 0.68% (12,810/1,895,829) to 0.12% (2379/2,017,921) (*P*<.001), and the degree of patient satisfaction was improved. Monthly outpatient visits were increased by 0.98% (3192/325,710) to 13.09% (42,939/328,032) compared with the same period the preceding year (*P*=.02).

**Conclusions:**

Smartphone-based triage significantly reduces the wrong registration rate caused by patient Web-based appointment registration and improves the degree of patient satisfaction. Thus, it is worth promoting.

## Introduction

### Background

Medical systems worldwide face clinical, administrative, regulatory, and financial strains that require them to increase performance while reducing costs [1,2]. Hospitals often have a limited number of support staff responsible for patient registration and for taking medical history [3]. Registration by medical staff can create a bottleneck that increases waiting time and, thus, the likelihood of registration errors [4-6].

The National Health and Family Planning Commission of the People’s Republic of China issued the Action Plan for Further Improving Medical Care Services in 2015, requiring appointment rates in tertiary hospitals to be at least 50% by the end of 2017 [7]. Therefore, hospitals began to implement appointment systems, and some of them implemented full appointment systems for nonemergency registration. The appointment systems alleviate the difficulty of temporary registration to a certain extent but may lead to wrong registration because of the limited medical knowledge of the patients. Song et al [8] reported that since the implementation of the full appointment system for nonemergency pediatric patients, the wrong registration rates by self-appointment and complaints from the children’s families cannot be neglected and that the most important problem faced by the patients is the selection of the correct department [8]. This is further complicated by increasingly subdivided departments [9]. Xuezhen et al [10] reported that the loss of time and money caused by wrong registration directly leads to patient dissatisfaction with hospital visits.

Wrong registration not only wastes the time of the patients but may also lead to disease aggravation and delayed treatment. Lewis et al [11] proposed an effective clinical decision support tool to perform prehospital diagnosis and triage of ruptured aortic aneurysms accurately. Adapting such algorithms into smartphone apps that are used to make appointment registration could help decrease the error rates. It is feasible to use smartphone apps to report a dental emergency [12] and burns [13]. Previous studies mainly reported the use of smartphones for emergency triage and referral for some diseases [14-16], but the added value of using smartphone-based triage for regular appointments remains undefined. The most concerning aspect of adequate appointment registration is that the parents’ level of information regarding their child’s illness is limited [17]. Mobile health is a promising app for appointment registration [14]. WhatsApp (WhatsApp Inc) helps reduce unnecessary referrals and outpatient visits [15]. Smartphone-based pulse oximetry has been used to evaluate pediatric patients without hypoxia [16]. Reduction in waiting time from referral to first visit for community outpatient services might contribute to better health outcomes [18]. Therefore, investigating smartphone-based triage is critical in the modern era where the smartphone is becoming a central actor in everyday life.

In many clinics in China, the patients now have the option to make Web-based appointments, but doing so according to their own judgment may lead to wrong registration and delayed medical services. On the basis of the above, we hypothesized that smartphone-based triage in outpatient services is superior to Web-based self-appointment registration guided by the medical staff.

### Objectives

Therefore, this study aimed to investigate the effect of smartphone-based triage on outpatient visits compared with Web-based self-appointment registration, the rate of wrong registration made by the patients, the degree of patient satisfaction, and the effects on outpatient visits. This study provides a reference for improving outpatient appointment registration.

## Methods

### Study Design and Subjects

This was a cross-sectional study of patients who made appointments with the outpatient department of Guangzhou Women and Children’s Medical Center between April 2018 and March 2019. This medical center is one of the hospitals that implemented a full appointment system for nonemergency appointment registration. There are more than 10,000 outpatient visits daily, and smartphone appointments among these outpatient visits accounted for a total of 77.15% (8692/11,266).

The inclusion criteria were that the participant (1) had records available during the study period in the hospital information system, (2) used a smartphone to book the appointment, and (3) had good communication skills and consented to participate. The exclusion criteria included (1) incomplete registration or appointment data or (2) multiple visits during the study period.

### Smartphone App

Our hospital and Shenzhen Tencent Technology jointly developed and implemented smartphone-based triage based on symptoms recorded since October 2018. At the time of appointment registration, the option of selecting basic symptoms was added. Patients or family members can select symptoms based on disease conditions, which then accordingly guide them in selecting the adequate departments and doctors to visit. The best department can be selected according to different symptoms in the registration symptom page. In addition, the best route guidance can be provided based on precise, intelligent navigation within the hospital.

### Patient Grouping

The patients recruited from April to September 2018 made Web-based appointment registration without the smartphone system, whereas from October 2018 to March 2019, outpatients started using the smartphone app for Web-based appointments.

### Indexing Process for Smartphone Self-Appointment System

**Figure 1:**
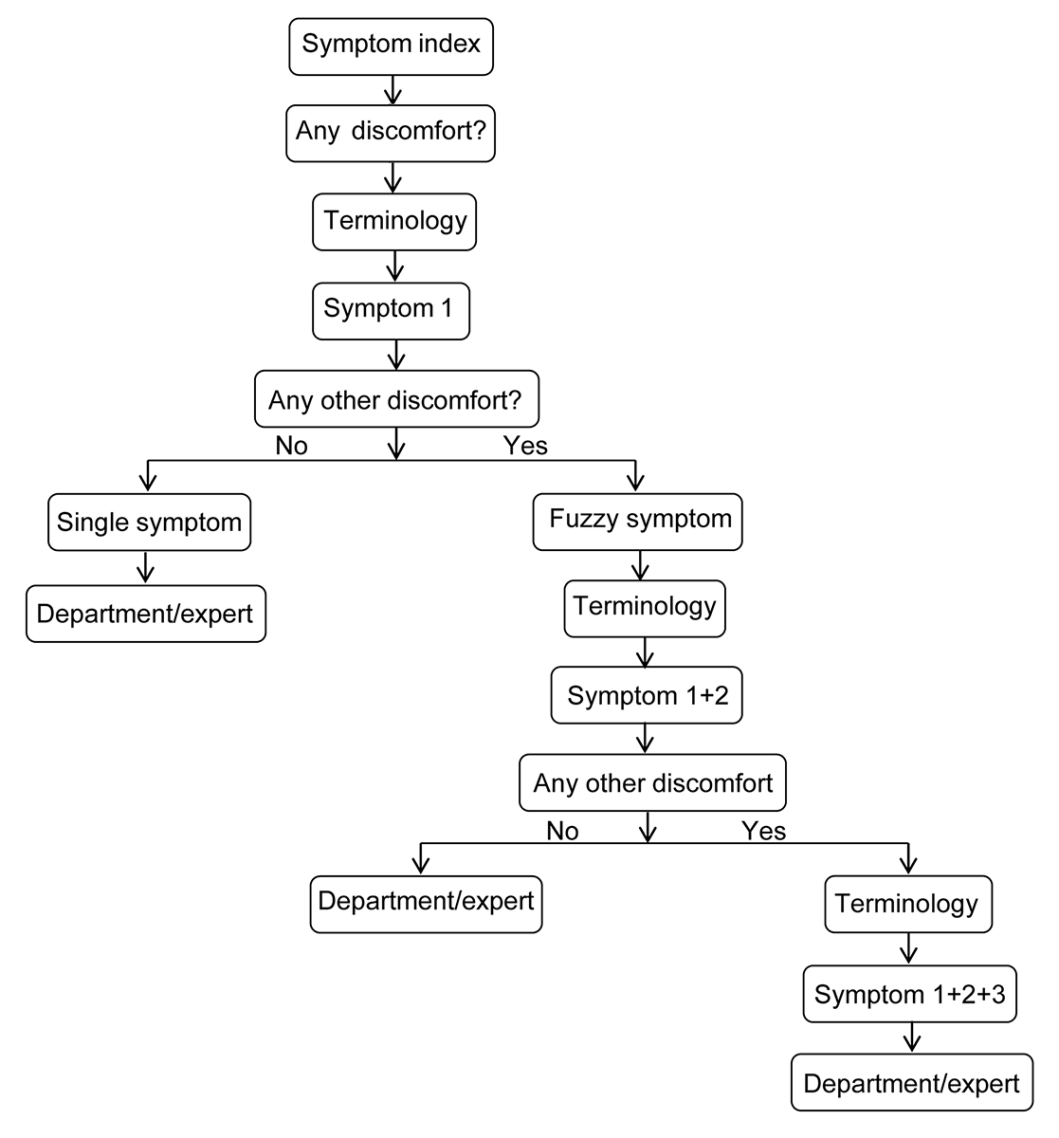
Self-appointment symptom index chart.

Disease indexing was performed by the patients. If the patient had a confirmed disease, the app immediately directed the patient to the appropriate department (Figure 1; Multimedia Appendix 1 shows screenshots of the app). If there was no confirmed diagnosis, the app asked a series of questions based on symptoms, discomfort, and terminology and then suggested the suitable departments to the patients. The app is completely automated.

### Data Collection

Smartphone-based triage had storage and memory functions. The medical staff could directly obtain the content from the background system and retrieve outpatient visits from the hospital operation and decision support system (business intelligence system). The degree of satisfaction regarding the accuracy of smartphone-based triage was extracted from the degree of satisfaction survey, which spanned the period from 6 months before smartphone-based triage to 6 months thereafter.

Patients in each department of Guangzhou Women and Children’s Medical Center were enrolled. Random sampling was performed for selection, and assessment was carried out by 55 investigators who underwent unified training. The degree of satisfaction survey was performed on site at the hospital. According to the sample size, the survey of 1850 cases indicated that the interviewees were representative. General satisfaction was the first-level indicator. In addition, there were 6 second-level indicators, including general impression, service attitude, service quality, hospital environment, price perception, and medical ethics. Furthermore, 31 third-level indicators were assessed (Multimedia Appendix 2). The responses to third-level indicators were categorized using a 5-point Likert scale. The answers were coded from 1 to 5: 5-very satisfied, 4-satisfied, 3-neutral, 2-unsatisfied, and 1-very unsatisfied. The current internationally recognized method for calculating the satisfaction degree based on public opinion was adopted for evaluating each of the third-level indicators [19,20]: 100, *very satisfied*; 80, *quite satisfied*; 60, *neutral*; 40, *dissatisfied*; and 20, *very* *dissatisfied*. *Unclear* was assigned for missing values, which were excluded from the final analysis. A single indicator was calculated based on weighted calculation: degree of satisfaction = proportion of very satisfied × 100 + proportion of relatively satisfied × 80 + proportion of generally satisfied × 60 + proportion of not satisfied × 40 + proportion of very dissatisfied × 20. The overall degree of satisfaction was calculated by weighing each indicator score [19,20]. General satisfaction was calculated by summing the scores of all second-level indicators after multiplying them by their corresponding weights: general impression (5%), service attitude (15%), service quality (20%), hospital environment (10%), price perception (20%), and medical ethics (30%) [19,20].

### Statistical Analysis

SPSS 23.0 (IBM) was used for data analysis. Queries and outpatient visits of smartphone-based triage were expressed as frequencies. The proportion of queries and inquiry content was expressed as a percentage. A comparison of wrong registration and the degree of satisfaction was analyzed by the Student *t* test. *P*<.05 was considered statistically significant.

### Ethical Considerations

The ethics committee of Guangzhou Women and Children’s Medical Center (approval number SFE-KL-28201) approved this study. Patients or their guardians provided signed informed consent.

## Results

### Effect of Smartphone-Based Triage

The average daily numbers of smartphone-based triage used for the 6 consecutive months were 2359, 1917, 1956, 1961, 3067, and 3643, accounting for 22.23% (2359/10,610), 16.37% (1917/11,717), 16.15% (1956/12,112), 23.20% (1961/8453), 26.09% (3067/11,754), and 29.46% (3643/12,366) of all outpatient visits, respectively. The average number of daily queries for the whole study period was 2730, which was equal to the workload of at least nine experienced precheck triage nurses and hospital guiding staff. The numbers of successful daily registrations after smartphone-based triage for the 6 consecutive months were 1437, 1101, 1130, 1101, 1736, and 2081, accounting for 60.92% (1437/2359), 57.43% (1101/1917), 57.77% (1130/1956), 56.14% (1101/1961), 56.60% (1736/3067), and 57.12% (2081/3643) of all queries, respectively, and 13.54% (1437/10,610), 9.40% (1101/11,717), 9.33% (1130/12,112), 13.02% (1101/8453), 14.77% (1736/11,754), and 16.83% (2081/12,366), respectively, of all daily outpatient visits in the respective months (Table 1).

**Table 1:** Queries of smartphone-based triage, successful registrations after query, and proportions of outpatient visits.

Time (months)	Daily outpatient visits	Proportion of daily queries to outpatient visits, n (%)	Proportion of successful daily registrations after query to outpatient visits, n (%)
1	10,610	2359 (22.23)	1437 (13.54)
2	11,717	1917 (16.37)	1101 (9.40)
3	12,112	1956 (16.15)	1130 (9.33)
4	8453	1961 (23.20)	1101 (13.02)
5	11,754	3067 (26.09)	1736 (14.77)
6	12,366	3643 (29.46)	2081 (16.83)

### Reduction in Wrong Registrations

The number of wrong registrations during the 6 months before smartphone-based triage was 12,810, accounting for 0.68% of all outpatient visits (1,895,829), that is, 2135 (SD 37) wrong registrations recorded monthly. This number was significantly reduced to 2379 after smartphone-based triage, accounting for 0.12% of all outpatient visits (2,017,921), that is, 396 (SD 17) wrong registrations recorded monthly (95% CI 1701.362-1771.68; *P*<.001).

### Improved Degree of Satisfaction of the Outpatients

A total of 7400 questionnaires were released and 7188 were collected, indicating an effective recovery rate of 97.14%. The questionnaire survey data are shown in Table 2. A comparison of the first and second quarters after smartphone-based triage with pre–smartphone-based triage showed a significant improvement in the degree of patient satisfaction (*P*<.001; Table 3).

**Table 2:** Basic information of the degree of patient satisfaction before and after smartphone-based triage.

Item and condition		Second quarter before triage	First quarter before triage	First quarter after triage	Second quarter after triage
**Valid questionnaires**					
	Copies	1832	1838	1802	1716
**Sex, n (%)**					
	Female	970 (52.95)	1112 (60.50)	1116 (61.93)	1065 (62.06)
	Male	862 (47.05)	726 (39.50)	686 (38.07)	651 (37.94)
**Payment, n (%)**					
	Self-paying	1280 (69.87)	1141 (62.08)	1168 (64.82)	1067 (62.18)
	Medical insurance	454 (24.78)	623 (33.90)	563 (31.24)	579 (33.74)
	Public expense	98 (5.35)	74 (4.02)	71 (3.94)	70 (4.08)
**Residence, n (%)**					
	Local	1290 (70.41)	1317 (71.65)	1300 (72.14)	1315 (76.63)
	Other city in local province	466 (25.44)	389 (21.17)	372 (20.64)	323 (18.82)
	Other province	76 (4.15)	132 (7.18)	130 (7.22)	78 4.55)
**Sample classification, n (%)**					
	Patient himself	336 (18.34)	443 (24.10)	527 (29.25)	501 (29.20)
	Relatives and friends	1496 (81.66)	1395 (75.90)	1275 (70.75)	1215 (70.80)
**Educational level, n (%)**					
	Below junior middle school	243 (13.26)	296 (16.10)	276 (15.32)	229 (13.34)
	High school or technical secondary school	432 (23.58)	432 (23.50)	421 (23.36)	340 (19.81)
	Junior college or undergraduate	1073 (58.57)	1028 (55.93)	1022 (56.71)	1062 (61.89)
	Master degree or above	84 (4.59)	82 (4.47)	83 (4.61)	85 (4.96)

**Table 3:** Comparison of satisfaction level before and after smartphone-based triage.

Time	After smartphone-based triage, mean (SD)	Before smartphone-based triage, mean (SD)	*P* value	Odds Ratio (95% CI)
First quarter	87.05 (3.49)	85.77 (3.71)	<.001	1.059(0.482-2.327)
Second quarter	87.60 (3.32)	85.27 (3.26)	<.001	0.773(0.342-1.747)

### Degree of Satisfaction Regarding the Accuracy of Smartphone-Based Triage

The survey conducted in 1850 patients about the degree of satisfaction regarding the accuracy of smartphone-based triage showed that 1601 patients were satisfied (88.68%), 144 were neutral (7.98%), 60 were dissatisfied (3.34%), and 45 were missing.

### Proportions of Inquiries in the Content of Smartphone-Based Triage

For patients inputting their diseases and symptoms in smartphone-based triage, those waiting for disease judgment accounted for 48,596 (57.68%), those looking for a doctor accounted for 14,184 (16.84%), those in the inquiry process accounted for 7260 (8.62%), those with other questions accounted for 6712 (7.97%), those inquiring for examination accounted for 3994 (4.74%), and those looking for the department accounted for 3502 (4.15%).

### Top 10 Queries of Disease Recommendation

The top 10 queries were associated with unspecified-site acute upper respiratory tract infection; other infectious diseases and unspecified etiologies, gastroenteritis, and colitis, other and unclear pyrexia, acute nasopharyngitis cold, influenza without labeled virus, cough, bronchitis unspecified as acute or chronic, pneumonia with unspecified pathogen, hand-foot-and-mouth disease, chronic disease of tonsils and adenoids.

### Top 10 Queries of Department Recommendation

The top 10 queries based on department recommendations were associated with the deputy director of outpatient internal medicine, attending internal medicine department, attending respiratory outpatient, attending auricle deformity department, chief of pediatric respiratory department, attending otolaryngology department, chief of otolaryngology department, attending dermatology department, deputy director of dermatology department and attending outpatient department of gastroenterology.

### Changes in Outpatient Visits Before and After Smartphone-Based Triage

The outpatient visits were increased by 0.98% (3192/325,710) to 13.09% (42,939/328,032) compared with the same period last year, and monthly outpatient visits after 6 months of smartphone-based triage were 328,902, 351,518, 375,458, 236,690, 364,382, and 370,971. Compared with the outpatient visits in the same period last year, the outpatient visits were increased by 0.98% (3192/325,710), 4.40% (14,815/336,703), 9.22% (31,695/343,763), 5.22% (11,742/224,948), 8.23% (27,708/336,674), and 13.09% (42,939/328,032), after smartphone-based triage (95% CI 6525.769-41,544.565; *P*=.02).

## Discussion

### Principal Findings

Human-based patient registration requires money, time, and human resources and often represents a bottleneck for patient registration [1-6]. In China, where the health care system is based on specialists rather than generalists, the patients now have the option to make Web-based appointments, but doing so according to their own judgment may lead to wrong registration and delayed medical services. This study investigated the effect of implementing smartphone-based triage in the outpatient department compared with Web-based self-appointment registration, smartphone-based triage queries, rate of successful registration after query, rate of wrong registration, degree of patient satisfaction, and the top 10 recommended diseases and departments. The results indicated that smartphone-based triage has significant effects on appointment registration and is well accepted by the patients and family members, reduces wrong registration caused by patient Web-based appointment registration, and improves the degree of patient satisfaction.

This showed that patients could use smartphones to consult the doctor anytime from anywhere to be guided to the right department, thereby optimizing the visit process, reducing the wrong registration rate, and improving the degree of patient satisfaction. De Bruin et al [21] confirmed that a smartphone-based triage app was well accepted by the patients.

Previous studies reported improved effectiveness of patient registration. Nogueira et al [22] proposed the FAST-ED app for improving smartphone-based triage in acute ischemic stroke patients, and Uthoff et al [23] reported the use of smartphones in the early discovery of oral cancer lesions. Tayfur and Afacan [24] showed that the patients could perform a smartphone-based triage evaluation before seeing a doctor. Verzantvoort et al [25] stated that smartphone-based triage plays an important role in patients who are consulting doctors. Tian et al [26] used a simulation model to evaluate a mobile-based system for supporting emergency evacuation decision making. Gupta et al [27] reported technicians using a store-and-forward telemedicine device to screen for ear problems. Astarcioglu et al [28], Borve et al [29], Dahlen Gyllencreutz et al [30], and Urner et al [31] investigated the use of smartphones in skin cancer referral, time-to-reperfusion in ST-segment elevation myocardial infarction undergoing inter-hospital transfers, teledermoscopy images acquired in primary health care and hospital settings, and accuracy in triage imaging of human papillomavirus–positive women, respectively. All these studies showed that the use of smartphone-based triage improved the wrong registration rates and patient satisfaction in specific patient populations. This study is supported by those studies and provides a higher degree of generalization as all women and pediatric patients were included, irrespective of their disease, and the department consulted. Nevertheless, those previous studies did not report how many outpatients were willing to participate in the clinical study. In contrast, this study demonstrated that queries associated with smartphone-based triage were significant, and the triage was well accepted by patients.

### Strengths and Limitations

A major advantage of the system used in this study was that no special smartphone requirement was set, and registration and smartphone-based triage could be followed after the patient had logged into the official WeChat account. Resources should be effectively allocated while using information systems [21]. In this study, smartphone-based triage took advantage of information technology to replace most of the work of routine field manual preinspection and triage by clinical service staff, effectively allocating resources. Not all patients used the smartphone app for appointment registration, but many of those who used the app did not require any assistance for the selection of the right department. This system could save time and energy of the support staff and effectively improve the utility of the precious medical resources.

Our hospital is specialized in pediatrics and woman health care; hence, more than 70% of outpatient visits were in the pediatric department. Nevertheless, the study showed that after the implementation of smartphone-based triage, the total outpatient visits increased compared with the previous year. The exact reasons for this increase remain unknown and deserve further investigation.

This study has limitations. The study site was limited to 1 hospital, and the collected data and indicators were limited. There was no randomization, and there could be a temporal bias. Multicenter trials with several indicators are required to confirm the findings. In addition, the app assessed in this study was specific to countries with a health system centered on hospital consultations by specialists, which is not the case in several other countries (eg, in Europe and America), where the patients consult a general practitioner who manages almost all diseases and refers to the proper specialists when needed. The questionnaire was not validated. Nevertheless, the 5-point Likert scale was used in this study. Finally, as the use of technology is often dependent upon age, financial resources, and education, future studies should examine the influence of those factors to optimize the use of the smartphone app for various populations of patients.

### Conclusions

In conclusion, smartphone-based triage significantly improves the rate of wrong patients’ Web-based appointment registration and improves the degree of patient satisfaction. It allocates expert resources more effectively, saving manpower for prediagnosis, triage, and patient guiding. Thus, such a smartphone approach is worth promoting. Future studies could examine its use by medical staff for inpatient management and transfer among departments. It could also be examined in general practitioners-centered settings.

## Appendix

Multimedia Appendix 1 Screenshots of smartphone-based triage.

Multimedia Appendix 2 Satisfaction questionnaire.
