# Supplementary figures and images for: The Use of Smartphone-Based Triage to Reduce the Rate of Outpatient Error Registration: Cross-Sectional Study
Source: JMIR Mhealth Uhealth. 2019 Nov 11;7(11):e15313. doi: 10.2196/15313 (PMC6878102; doi:10.2196/15313)

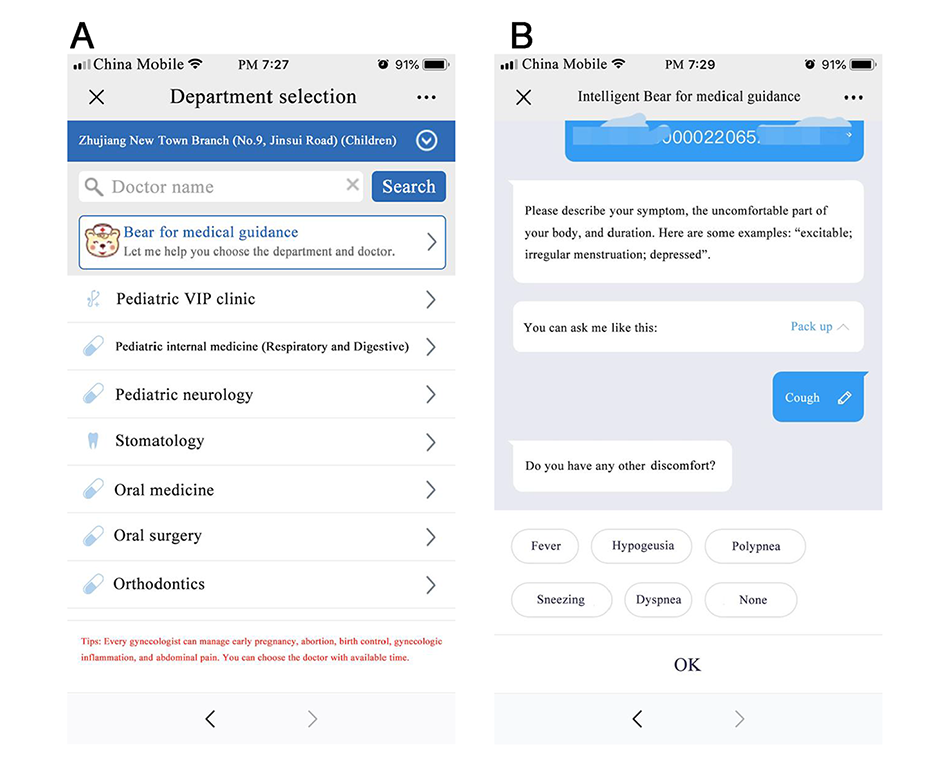

Supplement: Multimedia Appendix 1 [file mhealth_v7i11e15313_app1.png]
